# Impact of water on the fracture behavior of an advanced lithium disilicate

**DOI:** 10.7717/peerj.21170

**Published:** 2026-04-28

**Authors:** Yuqing Lu, João Paulo Mendes Tribst, János Kodolányi, Cornelis J. Kleverlaan, Albert J. Feilzer, Amanda Maria de Oliveira Dal Piva

**Affiliations:** 1 Reconstructive Oral Care, Academic Centre for Dentistry Amsterdam (ACTA), Universiteit van Amsterdam and Vrije Universiteit, Amsterdam, Netherlands; 2 Dental Materials Science, Academic Centre for Dentistry Amsterdam (ACTA), Universiteit van Amsterdam and Vrije Universiteit, Amsterdam, Netherlands

**Keywords:** Glass ceramics, Lithium disilicate, Fracture, Fatigue, Flexural strength

## Abstract

**Objective:**

To investigate the influence of water on the monotonic and fatigue strength of a lithium disilicate glass-ceramic.

**Methods:**

Ninety bar-shaped specimens (1.0 × 1.0 × 12.0 mm) were prepared from Advanced Lithium Disilicate (CEREC Tessera; Dentsply Sirona). Half of the specimens were stored in deionized water (W) at 37 °C for 30 days, while the other specimens stayed dry (D). A three-point bending test was carried out in a dry/wet environment (subgroup d/w) to determine monotonic strength. A stepwise fatigue test was conducted for dry-stored specimens in a dry environment (Dd) and wet-stored specimens in water (Ww).

**Results:**

For monotonic strength, the testing environment had a significant effect (*p* < 0.001), whereas the storage environment did not (*p* = 0.054). Testing in water generated a lower monotonic strength (Dw: 243 ± 35 MPa; Ww: 250 ± 57 MPa) than in a dry environment (Dd: 324 ± 74 MPa; Wd: 376 ± 60 MPa). Wet storage combined with testing in water exhibited similar fatigue strength (Ww: 148 ± 39 MPa) to the group without water intervention (Dd: 152 ± 36 MPa).

**Conclusions:**

While storage in 37 °C water for 30 days causes ion release from lithium disilicate, it does not decrease its monotonic strength. The compressive loading in water resulted in a degradation of around 30%. The fatigue protocol in this study resulted in about 50% of the initial strength regardless of testing conditions.

## Introduction

By the early 2020s, lithia-based glass-ceramics and zirconia have emerged as the leading restorative ceramic materials (Lubauer et al., 2022; Zhang et al., 2023; Chen et al., 2021). Lithia-based glass-ceramics, particularly lithium disilicate, are known for their exceptional aesthetics and satisfactory resistance to occlusal forces. Lithium disilicate, the most prominent type of lithia-based glass-ceramics, features a distinctive microstructure with interlocking needle-like crystals within a glass matrix. This structure promotes crack deflection and stopping, enhancing the material’s toughness and strength (Kelly et al., 2017; Al-Johani et al., 2024; Lubauer, Lohbauer & Belli, 2023a; Gonzaga et al., 2011).

The first available lithium disilicate block for the milling process was IPS e.max^®^ CAD (Ivoclar) in a pre-crystallized form with a blue appearance. After milling, the final crystallization process should be performed, where the platelet-like Li_2_SiO_3_ (lithium metasilicate) crystals grow into interlocked rod-like Li_2_Si_2_O_5_ (lithium disilicate) particles in the glass matrix (Phark & Duarte, 2022). Currently, with the growing need for chairside restoration, alternative lithium disilicate blocks are available on the market, such as the Advanced Lithium Disilicate (CEREC Tessera™, Dentsply Sirona), which requires only a fast-firing cycle (4 min 30 s) instead of a conventional crystallization process. However, unlike the proven long-term clinical success of the conventional IPS e.max^®^ CAD (Lempel et al., 2023; Moura et al., 2023), scientific documents regarding its short or long-term behavior are relatively limited.

Since dental restorations are maintained and function within the moist environment of the oral cavity, the impact of water is a significant issue for the durability of restorative materials (Borges et al., 2009; Porto et al., 2019; Niem, Youssef & Wöstmann, 2020; Porto et al., 2019). Despite the hydrolytic reaction of glass is inconspicuous, prolonged water storage at specific temperatures can lead to ions release and might pose a potential impact on the material properties (Porto et al., 2019; Niem, Youssef & Wöstmann, 2020). On the other hand, the existence of water molecules is deleterious to the strength of glass. As the siloxane bonds of the crack tip in glass can be strained under stress, the glass fracture can be chemically assisted by the hydrolytic reaction (Freiman, Wiederhorn & Mecholsky, 2009; Wiederhorn & Bolz, 1970; Charles, 1958), causing crack propagation to initiate at a stress intensity below its fracture toughness. Ceramics are also susceptible to subcritical crack growth under masticatory loading, leading to failure at lower stress (Studart et al., 2007; Zhang et al., 2019). While it is acknowledged that water penetration reduces the lifetime of glass-ceramic restorations (Kelly et al., 2017; Zhang et al., 2019), the impact of water on the new crystal-reinforced dental glass-ceramics, especially regarding ion release and stress corrosion, are not yet fully understood.

Therefore, the aim of this study was to evaluate the influence of water storage and water presence during mechanical loading on the fracture behavior of the Advanced Lithium Disilicate. The fractured pieces were collected for fractography. Additionally, the ions released in the water after storage were analyzed using an inductively-coupled plasma mass spectrometer (ICP-MS).

## Materials and Methods

### Specimens’ preparation

**Figure 1:**
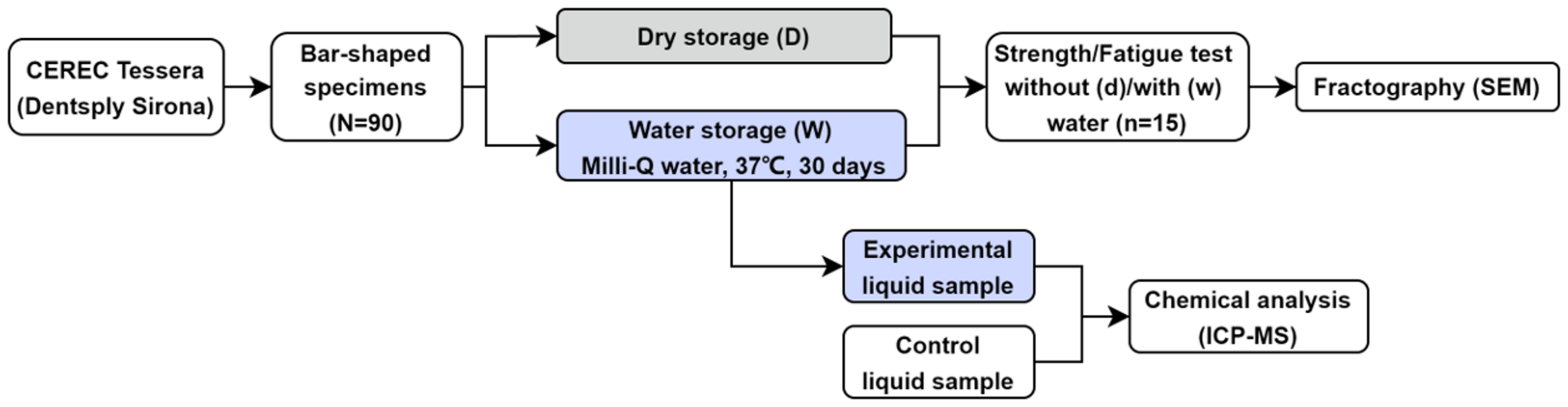
Flowchart of the study design.

A flowchart for this study design is presented in Fig. 1. Ninety bar-shaped specimens were cut from the Advanced Lithium Disilicate glass-ceramic (CEREC Tessera™; Batch number: 16013748; Dentsply Sirona, Hanau-Wolfgang, Germany) under water cooling using a diamond-coated saw in a precision cutting machine (Isomet 1000; Buehler, Lake Bluff, IL, USA). All the specimens were polished until the final dimension (1 × 1 × 12 mm) (Lu et al., 2024; Lu et al., 2023b; Lu et al., 2023a; Lu et al., 2023c; Machry et al., 2022) using SiC abrasive papers (Ecomet; Buehler Ltd., Evanston, IL, USA) with final grit sizes of P1200. They were cleaned in an ultrasonic bath with isopropyl alcohol for 5 min, followed by drying. All the specimens were submitted to a mandatory firing in a furnace (Programat P100; Ivoclar Vivadent, Schaan, Liechtenstein), according to the recommended protocol suggested by the manufacturer (closing time: 2 min, temperature gradient: 55 °C/min, holding temperature: 760 °C, holding time: 2 s, vacuum: off, long-term cooling: 0 °C/min, standby temperature: 403 °C). Before the mechanical tests, the specimens were randomly divided into two storage conditions: half of the specimens were stored in a sealed container filled with Milli-Q water (W) at 37 °C for 30 days (aqueous storage at body temperature), while the other fired specimens stayed dry (D) in air at room temperature (commonly used laboratory condition) for the same time.

### Monotonic strength test

**Figure 2:**
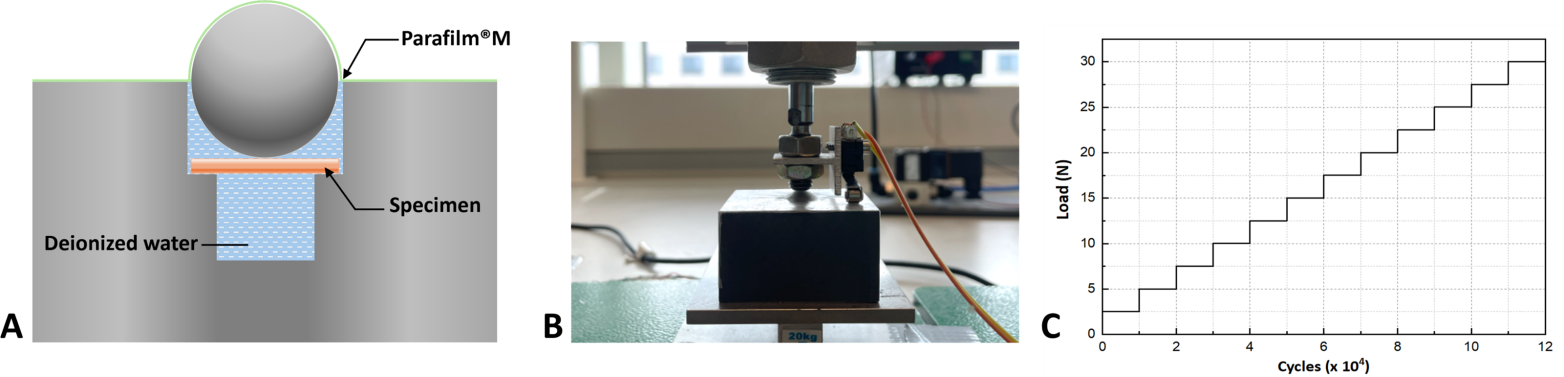
(A) The ball-in-hole device was used for the monotonic strength and fatigue tests. (B) The ACTA fatigue tester. (C) The stepwise loading protocol for the fatigue test.

For mechanical tests, a ball-in-hole testing device (Fig. 2A) with a span of 10 mm was used, which was described and verified in previous studies (Lu et al., 2024; Machry et al., 2022). Before and after each test, the testing device was carefully checked in case of possible damage, such as edge fracture and severe wear. The 3-point bending test was carried out using a universal testing machine (Instron 6022; Instron Limited, High Wycombe, UK) in a dry (d) testing environment or underwater, referred to as the wet (w) environment, both at room temperature. Sixty specimens from 4 groups (*n* = 15 per group) were evaluated, as shown in Table 1. The load was applied in the middle of the surface at 0.5 mm/min crosshead speed. Flexural strength (MPa) was calculated according to the following equation:

\begin{eqnarray*}{\sigma }_{\mathrm{u}}= \frac{3\mathrm{Pl}}{2\mathrm{b}{\mathrm{h}}^{2}} \end{eqnarray*}

**Table 1:** Groups’ distribution according to storage and testing environment, and mean values ± standard deviation of monotonic strength.

Group name	Storage	Testing environment	Monotonic strength (MPa)
Dd	Dry	Dry	324 ± 74^A^
Dw	Dry	Wet	243 ± 35^B^
Wd	Water	Dry	376 ± 60^A^
Ww	Water	Wet	250 ± 57^B^
*p*-value	0.054	<0.001	Interaction	0.140

**Notes.**

The same uppercase letter indicates no statistical significance.

where P is the maximum load (in N), l is the distance between the two supports (in mm), b represents the specimen width (in mm), and h is the specimen thickness (in mm). A two-way analysis of variance (ANOVA) was performed to assess the effects of water storage and testing environment on strength, followed by a Tukey test to compare the mean values across all groups.

### Stepwise fatigue test

The cyclic test was conducted using a fatigue testing machine (Figs. 2A & 2B, Fatigue Tester, ACTA, Netherlands) with the same bending set-up for two groups with or without water intervention (*n* = 15): dry-stored specimens in a dry environment (Dd) and wet-stored specimens in a wet environment (Ww), at room temperature. A piece of Parafilm^®^ was utilized to seal the device to prevent water leakage or evaporation during the cyclic test. A stepwise loading protocol was used (Fig. 2C), which started from 2.5 N and increased by 2.5 N in the next stress level until the specimen breaks. The maximum number of cycles in each level was 10,000 cycles at a frequency of 3 Hz. The load and number of cycles when the fracture occurred were recorded and analyzed using Kaplan–Meier. Maximum fatigue strength was calculated based on the specimen dimension and the following formula (Fraga et al., 2017):

\begin{eqnarray*}{\sigma }_{\mathrm{E}}={\sigma }_{0}+\Delta \sigma \left( {\mathrm{N}}_{\mathrm{fail}}/{\mathrm{N}}_{\mathrm{life}} \right) \end{eqnarray*}

where *σ*_0_ is the load level (N) before fracture occurs, and Δ*σ* is the load step (2.5N). N_fail_ is the recorded cycles at the step of fracture, while N_life_ is the defined total amount of cycles in each step (10,000 cycles). After a normality test, the maximum fatigue stress was submitted to a dependent student’s *t*-test.

### Fractography

The fractured specimens from the strength and fatigue tests were collected for fractography. Their fracture surfaces were primarily observed under optical microscope. Typical fractured pieces were selected and cleaned in a 90% ethanol-filled ultrasonic bath, followed by gold coating. A scanning electron microscope (SEM; EVO LS15; Carl Zeiss, Oberkochen, Germany) at different magnifications was used to identify the fracture origin.

### Inductively-coupled plasma mass spectrometer (ICP-MS)

To investigate the chemical interaction between the evaluated lithium disilicate and water, the liquid in the storage container was extracted for chemical analysis after all the specimens were removed. For control, a liquid sample in another container with only Milli-Q water (no lithium disilicate specimens) was also prepared after 30 days of storage under the same temperature. The two liquid samples were analyzed with an ICP-MS (Nexion 1000; Perkin Elmer, USA). The isotopes measured for quantitative analysis were ^6^Li, ^26^Mg, ^27^Al, ^29^Si, ^31^P, ^43^Ca, ^53^Cr, ^57^Fe, ^61^Ni, ^66^Zn, ^88^Sr and ^91^Zr. The amounts of released elements after 30-day storage were calculated based on their concentrations and the total volume of the evaluated liquids. The ion release amount per one mm^2^ was calculated according to the exposed surface area of the specimens.

## Results

**Figure 3:**
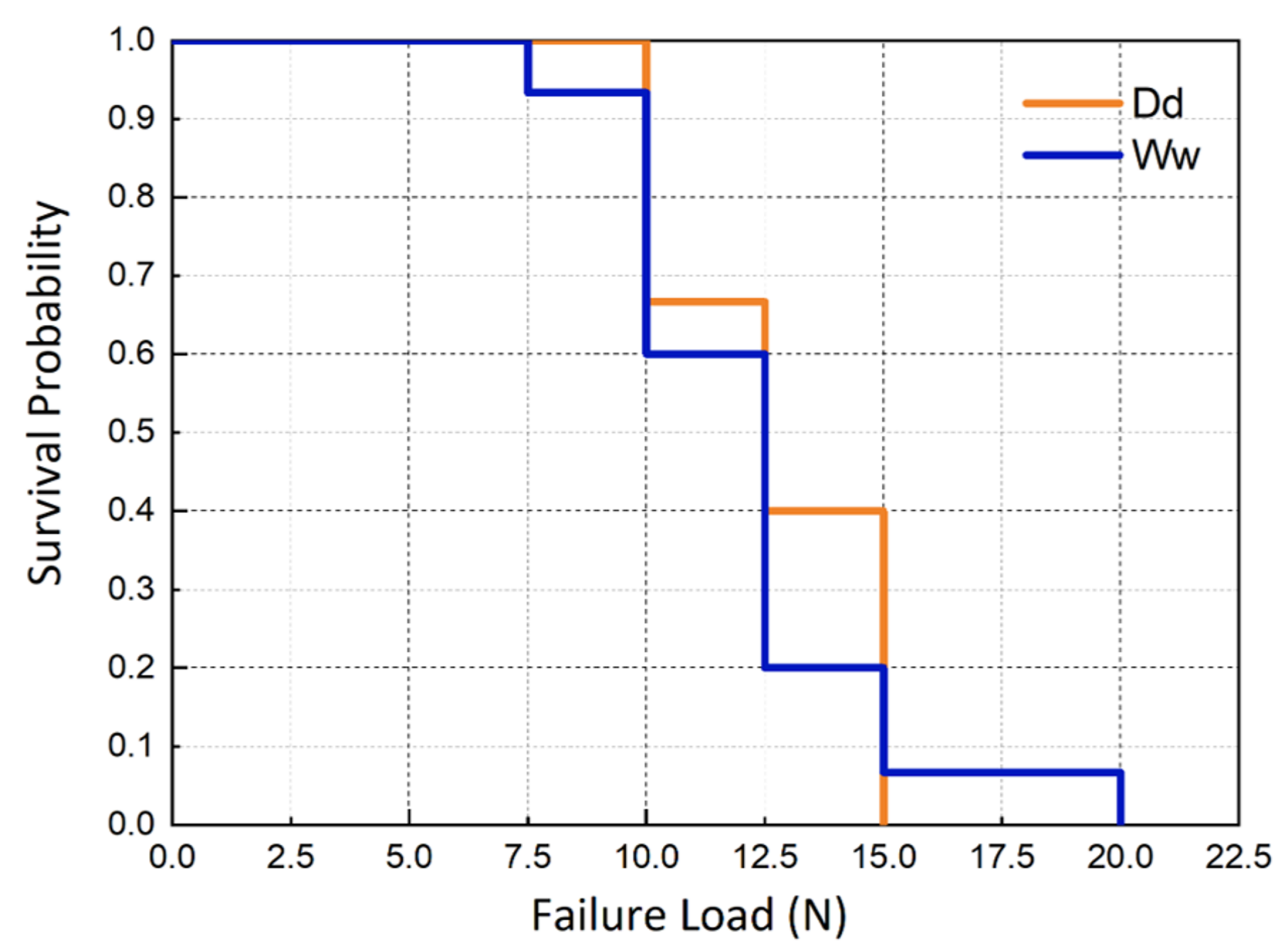
Survival curves of specimens tested in a wet environment after storage in water for 30 days (Ww) and a control group without water intervention (Dd).

The mean strength and standard deviation of each group are presented in Table 1. Two-way ANOVA showed a significant influence of the Testing Environment (*p* < 0.001) that the wet environment generated a significantly lower strength. However, the effect of water storage was not significant (*p* = 0.054), nor was the interaction Testing Environment*Water Storage (*p* = 0.14). For fatigue (Table 2), wet storage combined with a wet testing environment exhibited similar fatigue strength (Ww: 148 ± 39 MPa) to the group without water intervention (Dd: 152 ± 36 MPa), which is consistent with similar survival curves, as shown in Fig. 3.

**Table 2:** Mean values ± standard deviation of fatigue strength regarding water intervention.

Group	Water intervention	Fatigue strength (MPa)
Dd	None	152 ± 36^A^
Ww	Water storage + wet testing	148 ± 39^A^

**Notes.**

The same letter indicates no statistical significance in the column.

**Figure 4:**
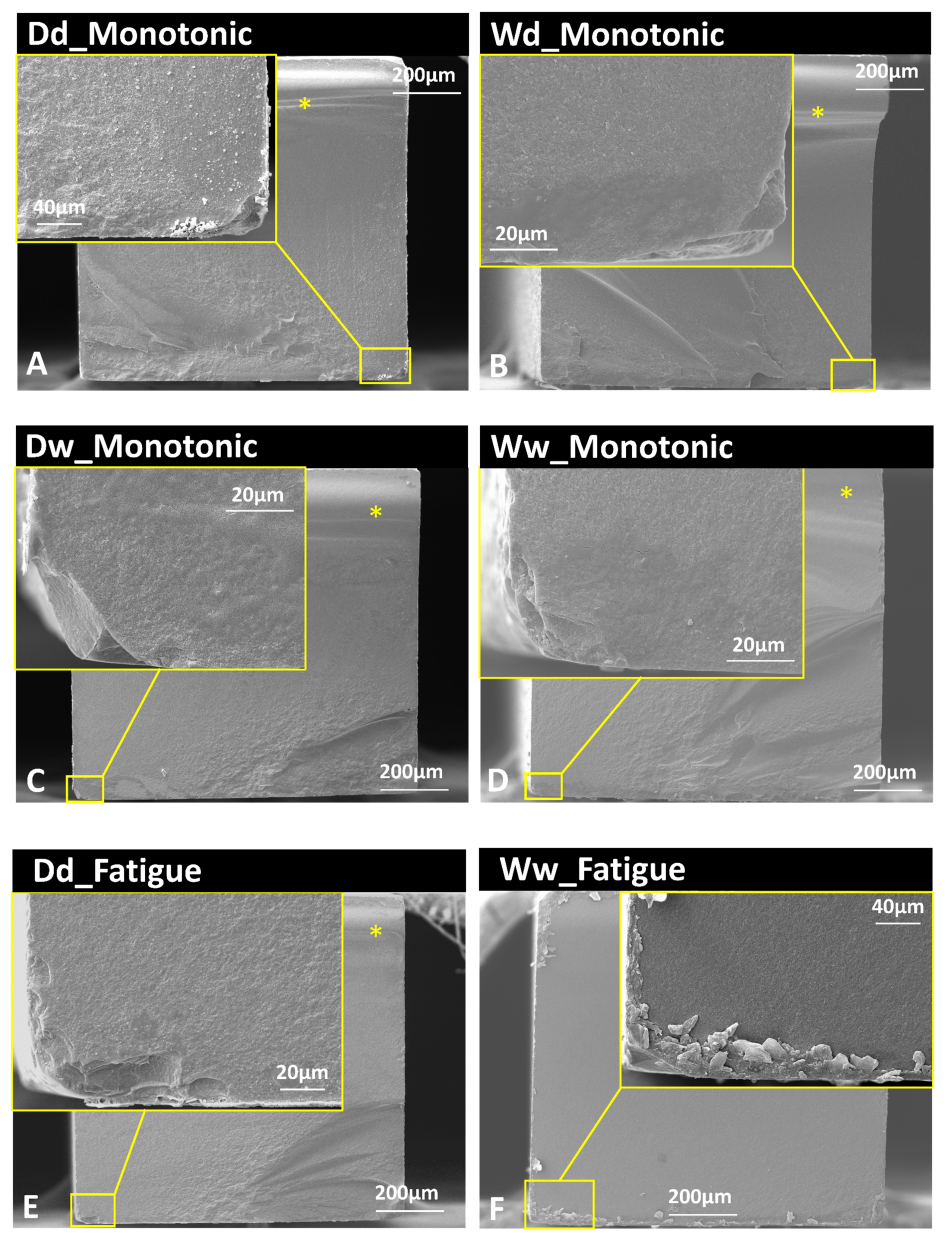
Fracture surfaces of the evaluated groups regarding mechanical tests (monotonic/fatigue) and water interventions: D/W-without/with water storage; d/w-mechanical test without/with water. The asterisk indicates the compression curl.

Figure 4 presents the SEM images showing that all the fractures initiated from the tensile side. However, different fracture origins were found for the monotonic and fatigue tests. For monotonic groups, the critical defects were mainly edge damage and surface flaws. For the fatigue groups, the direction of crack propagation suggests a potential fracture origin from the subsurface, which could be related to the subcritical crack growth.

The amounts of elements released in water after 30-day storage are summarized in Table 3. Lithium (Li) was the most dissolvable ion with the significantly highest detected amount, followed by minor amounts of phosphorus (P) and very slight amounts of silicon (Si) and chromium (Cr). None of the elements were detected in a significant amount for the control group (Milli-Q water only).

## Discussion

The purpose of this study was to investigate the influence of water storage and presence during mechanical testing on the fracture behavior of a lithium disilicate glass-ceramic. Based on the result of this study, water storage under 37 °C for 30 days did not degrade the monotonic strength of the evaluated lithium disilicate in the surface condition introduced in this study. Moreover, water storage followed by a wet testing environment does not affect fatigue strength, indicating that lithium disilicate restorations may perform reliably under functional fatigue loading. However, the monotonic strength was significantly reduced by around 30% in the wet testing environment compared to testing without water, highlighting the fracture risk for lithium disilicate restorations under high monotonic load/stress in the moist oral environment. This suggested that careful consideration is required when considering its use in patients with high bite forces or parafunctional habits.

While ceramics are usually considered to have high chemical stability, various ions, including Li, P, Si, and Cr, were found to be released during water storage. According to a previous study, the fired Advanced Lithium Disilicate consists of approximately 47 vol% residual glass, 38 vol% Li_2_Si_2_O_5_, 11 vol% Li_3_PO_4_, minor quartz and Virgilite (Lubauer et al., 2022). The residual glass is composed of SiO_2_, Li_2_O_,_ and other additives such as color agent Cr_2_O_3_. Among the detected elements, Li had the highest release amount, possibly from one or a combination of Li_2_Si_2_O_5_, Li_3_PO_4,_ and residual Li_2_O. In this study, the molar ratios of Li/Si and Li/P were considerably greater than the ratios in the chemical formula of the crystals, indicating that the majority of the lithium was released from Li_2_O in the residual glass. However, the dissolution of SiO_2_ isvery slight in comparison with Li_2_O, which can be attributed to the significantly higher bond strength of Si-O compared to Li-O (Lubauer, Lohbauer & Belli, 2023b). Consistent with this finding, it was found in another study (Esquivel-Upshaw et al., 2013) that lithium disilicate generates a higher weight loss than glaze material, in which lithium oxide is not one of the main components. Despite the detection of released ions, water storage for 30 days did not decrease the monotonic strength of the evaluated lithium disilicate, which is in accordance with previous findings that water storage for 7 days did not significantly influence the mechanical properties of glass-ceramics (Porto et al., 2019; Niem, Youssef & Wöstmann, 2020). Additionally, there seemed to be an increasing tendency in the average strength value from 324 MPa to 376 MPa after storage, although the effect of water storage was not significant (*p* = 0.054). Further research is needed to investigate the effect of longer storage periods in water on the strength and fatigue behaviour.

**Table 3:** Ion release in water per 1 mm^2^ surface area after 30 days of storage.

Element	Li	P	Si	Cr
Molar quantity (nM)	4.40	1.01 × 10^−1^	2.56 × 10^−3^	1.12 × 10^−5^

The wet testing environment showed a detrimental influence on the monotonic strength of the evaluated lithium disilicate, which is consistent with the well-known deleterious impact of water on glass. However, such degradation in lithium disilicate had a higher percentage of 30% in comparison with the previously reported 20% in glass (Baker & Preston, 1946). The mechanism behind the strength degradation in lithium disilicate could be more complex in comparison with pure glass, requiring further evidence for understanding. The toughening mechanism of the Li_2_Si_2_O_5_crystals was related to hierarchical deformation from the breakage of the Li-O bond to the final breakage of the Si-O (Deng et al., 2020). It remained unknown if water penetration at the crack tip could also facilitate the crack propagation in the Li_2_Si_2_O_5_crystals not only in glass matrix.

However, water intervention, including water storage and wet testing, did not result in a discernible impact on the fatigue strength of the evaluated lithium disilicate, which was rather unexpected. There are two possible explanations. Firstly, the effect of water impact could be stress-dependent: in the monotonic test, the applied stress was sufficient for cracks to propagate through the lithium disilicate crystal, which can be promoted by the hydrolysis reaction. However, for the fatigue test, there could be a critical threshold, below which the crack growth could be stopped at the crystals. Considering that the maximum stress in the fatigue test was approximately 50% of the monotonic test while the strength decrease by water was around 30%, the degradation by wet fatigue is probably dominated by cyclic loading. Secondly, subsurface crack growth was visible for the fatigue specimens, as exhibited in Fig. 4. The formation of these internal microcracks could be associated with structural inhomogeneities, leading to catastrophic fractures when they reach critical sizes, which is known as subcritical crack growth (Kelly et al., 2017). Due to the high densification of ceramic material, water could be difficult to penetrate these internal microcracks during mild stress loading. This is different from the situation during the monotonic test, where the fracture usually originated from the highly stress-concentrated surface defects that were exposed to water molecules.

One limitation of this study is that the saliva and diet of the patient affect the pH values of the oral environment, which can influence the ion release and mechanical properties of lithium disilicate (Esquivel-Upshaw et al., 2013; Hsu et al., 2020). Specifically, exposure to basic pH solutions leads to the breakdown of the silica network; acidic pH results in selective ion leaching from the glass matrix; at neutral pH, the two situations occur simultaneously (Esquivel-Upshaw et al., 2013). Additionally, as reinforced glass-ceramics contain crystalline compositions, the mechanism behind the ion release and its kinetics under various pH values could be more complicated than in pure glass. Moreover, the influence of aqueous environments with different pH values on the mechanical properties of lithium disilicate needs to be further investigated.

Another limitation of the present study is the fatigue results only reflect the combined influence of water storage and testing environment rather than isolating each factor independently. Although the presence of water did not show a significant impact on the cyclic tests of this study, it may affect results under different fatigue testing setup and protocols, with variations in test cycles, stress levels, and/or loading frequency. Meanwhile, there are a variety of available lithium disilicate ceramics or lithia-based glass-ceramics commercial products with differences in their chemical compositions and particle sizes, leading to differences in the resistance against crack propagation (Lubauer, Lohbauer & Belli, 2023b). Further studies will focus on comparing the effects of water on the fracture behavior of different lithium disilicate systems. Additionally, clinical lithium disilicate restorations are usually applied with a glaze layer to adjust color and improve surface quality. Therefore, the fracture could also initiate from defects within the glaze layer at the interface between the glaze and ceramic (Lu et al., 2023b; Lu et al., 2023a). Future study will investigate the water impact on the glazed lithium disilicate during monotonic and fatigue testing.

## Conclusion

Within the limitations of this study, it can be concluded that:

(1) Storage in 37 °C water for 30 days causes ion release from lithium disilicate, including lithium, phosphorus, silicon, and chromium.

(2) Water storage in this study did not decrease the monotonic strength of the evaluated lithium disilicate, while the testing in water degraded around 30% of the material’s strength.

(3) The fatigue testing setup and loading protocol in this study resulted in about 50% of the initial strength, whereas the water storage and testing environment did not affect the fatigue strength.

## Supplemental Information

10.7717/peerj.21170/supp-1 Supplemental Information 1 Raw data Sheet 1 presents montonic test data including specimens’ dimension and load. Sheet 2 showed the stepwise fatigue test data including step load and cycles when failures occured. Sheet 3 exhibits concentrations of elements (ions) detected in the storage water in comparison with control.

## Additional Information and Declarations

### Data Availability

The following information was supplied regarding data availability: The raw data is available in the Supplemental File.
